# Correction: Enciu et al. Is Complete Excision Always Enough? A Quality of Sexual Life Assessment in Patients with Deep Endometriosis. *Medicina* 2024, *60*, 1534

**DOI:** 10.3390/medicina60121965

**Published:** 2024-11-29

**Authors:** Raluca Gabriela Enciu, Octavian Enciu, Dragoș Eugen Georgescu, Adrian Tulin, Adrian Miron

**Affiliations:** 1 Clinical Hospital of Obstetrics and Gynecology “Prof. Dr. Panait Sârbu”, 060251 Bucharest, Romania; ralucagabrielasuba@gmail.com; 2 Medicover Endometriosis Center, 013982 Bucharest, Romania; 3 Discipline of General Surgery, Faculty of Medicine, “Carol Davila” University of Medicine and Pharmacy, 050474 Bucharest, Romania; dragos-eugen.georgescu@umfcd.ro (D.E.G.); adrian.tulin@umfcd.ro (A.T.); adrian.miron@umfcd.ro (A.M.)

## Missing Acknowledgement

In the original publication [1], the Acknowledgement “Publication of this paper was supported by the University of Medicine and Pharmacy Carol Davila, through the institutional program Publish not Perish” was not included. The authors state that the scientific conclusions are unaffected. This correction was approved by the Academic Editor. The original publication has also been updated.
